# *Escherichia coli* and *Staphylococcus* phages: effect of translation initiation efficiency on differential codon adaptation mediated by virulent and temperate lifestyles

**DOI:** 10.1099/vir.0.000050

**Published:** 2015-05

**Authors:** Ramanandan Prabhakaran, Shivapriya Chithambaram, Xuhua Xia

**Affiliations:** Department of Biology and Center for Advanced Research in Environmental Genomics, University of Ottawa, 30 Marie Curie, PO Box 450, Station A, Ottawa, Ontario K1N 6N5, Canada

## Abstract

Rapid biosynthesis is key to the success of bacteria and viruses. Highly expressed genes in bacteria exhibit a strong codon bias corresponding to the differential availability of tRNAs. However, a large clade of lambdoid coliphages exhibits relatively poor codon adaptation to the host translation machinery, in contrast to other coliphages that exhibit strong codon adaptation to the host. Three possible explanations were previously proposed but dismissed: (1) the phage-borne tRNA genes that reduce the dependence of phage translation on host tRNAs, (2) lack of time needed for evolving codon adaptation due to recent host switching, and (3) strong strand asymmetry with biased mutation disrupting codon adaptation. Here, we examined the possibility that phages with relatively poor codon adaptation have poor translation initiation which would weaken the selection on codon adaptation. We measured translation initiation by: (1) the strength and position of the Shine–Dalgarno (SD) sequence, and (2) the stability of the secondary structure of sequences flanking the SD and start codon known to affect accessibility of the SD sequence and start codon. Phage genes with strong codon adaptation had significantly stronger SD sequences than those with poor codon adaptation. The former also had significantly weaker secondary structure in sequences flanking the SD sequence and start codon than the latter. Thus, lambdoid phages do not exhibit strong codon adaptation because they have relatively inefficient translation initiation and would benefit little from increased elongation efficiency. We also provided evidence suggesting that phage lifestyle (virulent versus temperate) affected selection intensity on the efficiency of translation initiation and elongation.

## Introduction

Bacterial species and viruses need to replicate themselves rapidly in order to successfully compete against others. Translation is a key limiting factor in biosynthesis and microbial species typically evolve features to improve translation efficiency. Codon usage in *Escherichia coli*, *Salmonella typhimurium* and *Saccharomyces cerevisiae* strongly depends on the availability of their cognizant tRNA species (Ikemura, 1981a, b, 1982, 1992; Xia, 1998), especially in highly expressed genes (Coghlan & Wolfe, 2000; Comeron & Aguadé, 1998; Duret & Mouchiroud, 1999; Xia, 2007). Similarly, codon usage in bacteriophages (phages) is strongly shaped by the tRNA pool of their host (Chithambaram *et al.*, 2014a, b). Experimental modification to improve or disrupt codon adaptation generally leads to a predictable change in the protein production rate (Haas *et al.*, 1996; Ngumbela *et al.*, 2008; Robinson *et al.*, 1984; Sørensen *et al.*, 1989). In fact, gene-specific codon usage indices (Sharp & Li, 1987; Sun *et al.*, 2013; Wright, 1990; Xia, 2007) are excellent predictors of translation efficiency (Coghlan & Wolfe, 2000).

**Fig. 1.**
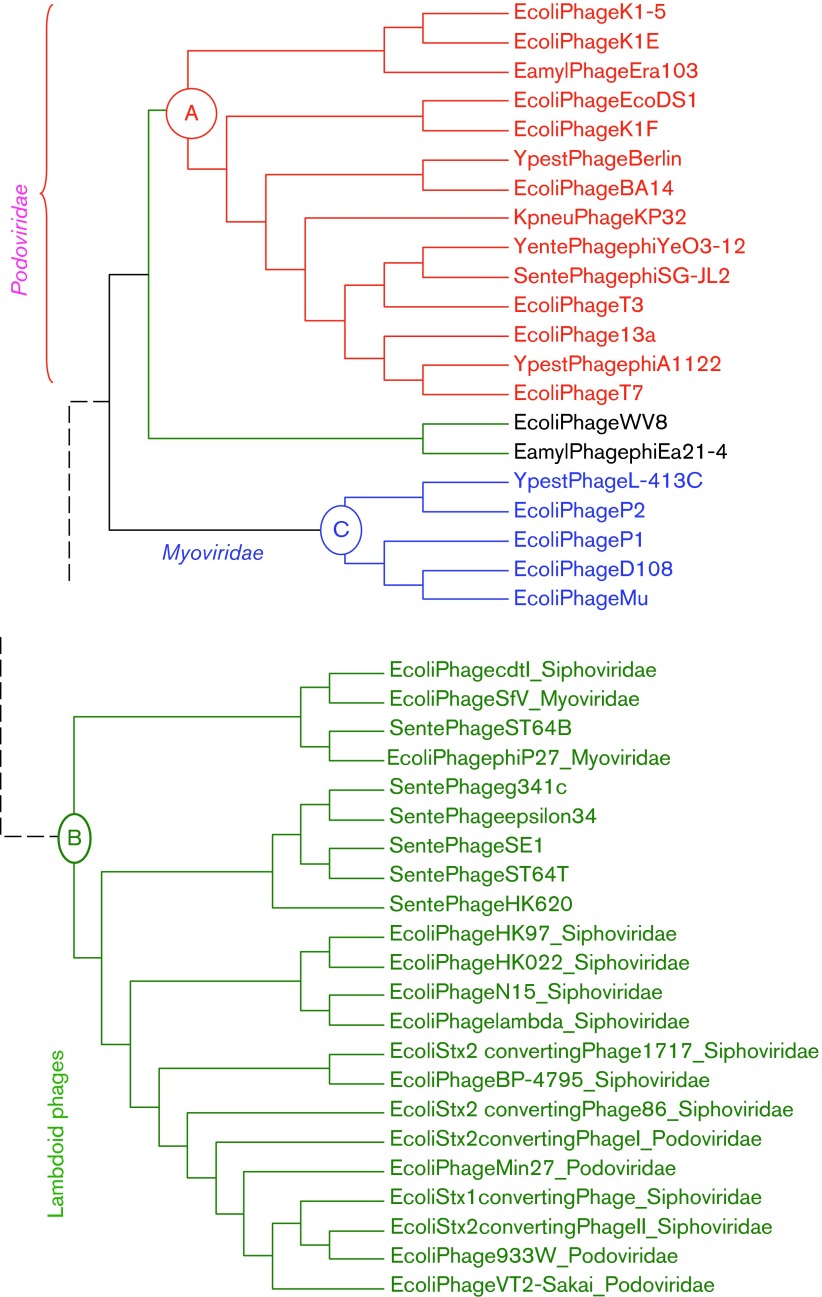
Partial phylogenetic tree showing two clades of phages (A and B), with Clade A exhibiting stronger codon adaptation to host *E. coli* than Clade B. Modified from Chithambaram *et al.* (2014b).

In this context it is puzzling that a large cluster of 16 *E. coli* lambdoid phages (Clade B in Fig. 1), consisting of 10 siphophages, four podophages and two myophages, exhibits poor codon adaptation in Y-ending codons in their protein-coding genes, whereas eight *E. coli* podophages in Clade A (Fig. 1) uniformly exhibit strong codon adaptation (Chithambaram *et al.*, 2014b). The same pattern remains if one measures codon adaptation by using the Codon Adaptation Index (CAI) (Sharp & Li, 1987) or its improved version (Xia, 2007) when *E. coli* highly expressed genes are used as a reference set, or by the index of translation elongation (*I*_TE_) that takes into account the effect of background mutation bias (Xia, 2014). Thus, genes in Clade B phages have significantly weaker codon adaptation than those in Clade A phages.

Three possible explanations for poor codon adaptation in Clade B phages to the host tRNA pool have been proposed but dismissed on the basis of empirical evidence (Chithambaram *et al.*, 2014a, b). The first invokes the differential presence of phage genome-encoded tRNA genes, which vary from zero to 20 in different *E. coli* phages (Chithambaram *et al.*, 2014a). A large number of phage-encoded tRNA genes would reduce the dependence of phage codon decoding on host tRNAs and allow the phage codon usage to deviate from host codon usage (Limor-Waisberg *et al.*, 2011; Prabhakaran *et al.*, 2014). Indeed, the degree of codon adaptation decreases with increasing number of phage-encoded tRNA genes (Chithambaram *et al.*, 2014a). It was also reported that selective enrichment of host tRNA by human immunodeficiency virus type 1 can also decrease the likelihood of the virus acquiring a codon usage similar to the host (van Weringh *et al.*, 2011). However, the difference in phage-encoded tRNA genes is minimal between the two clades in Fig. 1. Five Clade B phages (enterobacteria phages 933W, Min27, VT2-Sakai, and Stx2-converting phages II and 86) have three phage-encoded tRNA genes and one Clade B phage (enterobacteria phage ϕP27) has two phage-encoded tRNA genes. All other Clade B phages, as well as all Clade A phages, do not have phage-encoded tRNA genes. Those six Clade B phages carrying two or three tRNA genes do not have codon adaptation better or worse than other Clade B phages based on a *t*-test of the *I*_TE_ (Xia, 2014) between the two groups [*t* = 0.0879, degrees of freedom (d.f.) = 14, *P* = 0.9312, two-tailed test].

The second explanation attributes poor codon adaptation to lack of evolutionary time if phages have recently switched hosts. Take, for example, *E. coli* phage PRD1 which exhibits poor codon adaptation to the host (Xia, 2014). As the closest relatives of phage PRD1 all parasitize Gram-positive bacteria that have different codon usage from that of *E. coli*, phage PRD1 most likely has only recently switched to *E. coli* and consequently has had little time to evolve codon adaptation to the new *E. coli* host. However, this explanation is also inapplicable to the differential codon adaptation between Clade A and Clade B phages because both have diverse lineages parasitizing *E. coli* and should have evolved in the *E. coli* host for a long time. An associated possibility is that Clade B phages may have a more diverse host range than Clade A. If Clade B hosts happen to have diverse codon usage, then good codon adaptation to one host would mean poor codon adaptation to other hosts. Thus, parasitizing different hosts with different codon usage would interfere with codon adaptation to one particular host such as *E. coli*. However, we were not able to find conclusive evidence that Clade B phages have a more diverse host range than Clade A phages. Both clades can parasitize hosts such as *Yersinia pestis*, *Salmonella enterica* and *Klebsiella pneumonia*, in addition to *E. coli*. Furthermore, highly expressed protein-coding genes in all these four host species have almost identical codon usage. Thus, switching among these hosts should not interfere with phage codon adaptation to *E. coli*.

The third explanation invokes strand asymmetry and associated mutation bias often observed in circular microbial and mitochondrial genomes (Marín & Xia, 2008; Xia, 2012a, c). Highly expressed *E. coli* genes prefer CCU over CCC codons and UUC over UUU codons. However, phage CCY codons are mainly found in C-rich segments of the phage genome with over-represented CCC codons that are not preferred by *E. coli* highly expressed genes. Similarly, UUY codons are mainly found in T-rich genomic segments of the phage genome with over-represented UUU codons that are not preferred by *E. coli* highly expressed genes. However, whilst this explanation works well for ssDNA phages (Chithambaram *et al.*, 2014b), it does not seem sufficient to explain the poor codon adaptation in the dsDNA phages in Clade B relative to those in Clade A (Fig. 1).

Here, we proposed a hypothesis invoking differential translation initiation between the two clades of phages, based on the recent recognition that codon adaptation depends on translation initiation efficiency (Supek & Šmuc, 2010; Tuller *et al.*, 2010; Xia, 2014; Xia *et al.*, 2007). If translation initiation is highly efficient, then translation elongation will become rate-limiting and the selection for increasing translation efficiency will drive codon adaptation. If translation initiation is not efficient, then the selection for increasing translation efficiency will not reach codon usage because elongation is not rate-limiting. Thus, if translation initiation is more efficient in Clade A phages than in Clade B phages, then the selection for translation elongation efficiency will be stronger on Clade A phages than on Clade B phages, leading to differential codon adaptation.

To test the hypothesis that Clade A and Clade B phages have different translation initiation efficiencies, we need to measure the translation initiation efficiency. In bacterial species, translation initiation efficiency depends strongly on three factors: (1) the nature of the start codon (Hartz *et al.*, 1991; Ma *et al.*, 2002; O’Donnell & Janssen, 2001; Osterman *et al.*, 2013; Ringquist *et al.*, 1992), (2) the base-pairing potential and position of the Shine–Dalgarno (SD) sequence (de Smit & van Duin, 1994; Hui & de Boer, 1987; Olsthoorn *et al.*, 1995; Osterman *et al.*, 2013; Shine & Dalgarno, 1974), and (3) the stability of the secondary structure of sequences flanking the SD sequence and start codon (de Smit & van Duin, 1990, 1994; Milón *et al.*, 2012; Milón & Rodnina, 2012; Nivinskas *et al.*, 1999; Osterman *et al.*, 2013), with higher translation initiation generally associated with weaker secondary structure. dsDNA phages are known to have reduced secondary structure near the start codon (Zhou & Wilke, 2011), presumably to avoid having the SD sequence and start codon embedded in the secondary structure.

Pairing between the SD sequence and anti-SD (aSD) sequence on the small subunit ribosomal RNA is important for start codon localization (Hui & de Boer, 1987; Vimberg *et al.*, 2007), although such pairing is not always essential in translating *E. coli* messages (Fargo *et al.*, 1998; Melançon *et al.*, 1990) or in *Chlamydomonas reinhardtii* chloroplasts (Fargo *et al.*, 1998). Some leaderless genes with an AUG start codon can be translated efficiently in *E. coli* (Giliberti *et al.*, 2012; Krishnan *et al.*, 2010; O’Donnell & Janssen, 2002; Vesper *et al.*, 2011) or in the halophilic archaeon *Halobacterium salinarum* (Sartorius-Neef & Pfeifer, 2004). However, translation initiation of most *E. coli* genes appears to benefit from a well-positioned SD sequence, especially genes that follow the first gene in a multigene operon (Osterman *et al.*, 2013). In general, the effects of the SD sequence and the stability of the secondary structure flanking the SD sequence and start codon have become so well established that they serve as key design principles for computational tools optimizing translation initiation, such as RBSdesigner (Na & Lee, 2010), RBScalculator (Salis, 2011) and UTRdesigner (Seo *et al.*, 2013). As protein-coding genes in both Clade A and Clade B phages use AUG as the start codon, we tested the difference in the second and third factors between the two groups of phages. We predict that Clade A phage genes had stronger well-positioned SD sequences than those in Clade B phages and that Clade A phage genes also had weaker secondary structure in sequences flanking the SD and start codon than those in Clade B phages. These predictions were strongly supported by our empirical analysis of host and phage genomic sequences.

Given that Clade A phages exhibit better adaptation in translation initiation and elongation than Clade B phages, one would naturally ask if the former is under stronger selection for translation efficiency than the latter. One relevant observation is that all eight phage species in Clade A were virulent and all 16 phage species in Clade B were temperate (with a lysogenic phase). In contrast to virulent phages that are almost always engaged in translation once they enter the host cell, protein-coding genes in a prophage are not under any purifying selection and the evolutionary success of temperate phages does not necessarily rely on rapid biosynthesis. Thus, selection for more efficient translation may be stronger in the virulent phages than in the temperate phages, leading to more efficient translation initiation and better codon adaptation in the virulent (Clade A) phages than the temperate (Clade B) phages. This hypothesis is consistent with coliphages. To test its generality, we analysed phages infecting the Gram-positive *Staphylococcus aureus*, which has more sequenced phage genomes than any other Gram-positive bacterial species. The lifestyle hypothesis was consistent with the empirical evidence.

## Results

Our first objective was to explain why Clade A phages exhibited better codon adaptation to the *E. coli* host than Clade B phages and our hypothesis was that translation initiation was more efficient in the former than the latter so that codon adaptation would increase the protein production rate more in the former than in the latter. Our specific predictions were that: (1) the proportion of SD-containing genes (*P*_SD_) is higher in Clade A phages than in Clade B phages, (2) the length of SD–aSD pairing [mean number of consecutively matched sites (*M*_SD_)] should be closer to the optimal in Clade A phages than in Clade B phages, with the optimal SD length being 6 nt (Komarova *et al.*, 2002; Schurr *et al.*, 1993; Vimberg *et al.*, 2007), and (3) the minimum folding energy (MFE) in 40 nt upstream of the start codon (MFE_40nt_) and the MFE at sites from four sites upstream of the start codon to 37 sites downstream of the start codon (MFE_−4+37_) are less negative in sequences flanking the start codon in Clade A phages than in Clade B phages.

### Comparison of SD sequence features between Clade A and Clade B phages

*P*_SD_ was highly significantly greater in the eight Clade A phages (mean *P*_SD_ = 94.20 %) than in the 16 Clade B phages (mean *P*_SD_ = 68.27 %), as we had predicted (Table 1, *t*-test assuming unequal variances: *t* = 10.9900, d.f. = 21, *P*<0.0001, two-tailed test). We used the *t*-test with unequal variances because the two variances are significantly different from each other according to an *F*-test (*F* = 8.2400, d.f._numerator_ = 15, d.f._denominator_ = 7, *P* = 0.0045). However, a regular *t*-test assuming equal variance also strongly rejected the null hypothesis of equal *P*_SD_ between Clade A and Clade B phages (*t* = 8.3340, d.f. = 22, *P*<0.0001, two-tailed test).

**Table 1.** SD sequence features (*P*_SD_ and *M*_SD_) in Clade A and Clade B phages

Phage	GenBank accession no.	No. of coding sequences	*P*_SD_	*M*_SD_
**Clade A**				
T7	NC_001604	60	96.670	5.879
T3	NC_003298	55	90.910	6.020
K1F	NC_007456	43	88.370	5.921
K1E	NC_007637	62	95.160	5.763
K1-5	NC_008152	52	96.150	5.600
BA14	NC_011040	52	94.230	5.878
EcoDS1	NC_011042	53	94.340	6.140
13 a	NC_011045	55	96.360	5.943
**Clade B**				
VT2-Sakai	NC_000902	83	62.650	5.000
933W	NC_000924	80	70.000	5.036
λ	NC_001416	73	69.860	4.922
N15	NC_001901	60	80.000	5.000
HK022	NC_002166	57	56.140	4.875
HK97	NC_002167	61	68.850	5.024
ϕP27	NC_003356	58	67.240	5.359
SFV	NC_003444	53	69.810	4.676
Stx2-I	NC_003525	166	47.590	4.873
BP-4795	NC_004813	85	63.530	5.019
Stx1-phage	NC_004913	84	80.950	5.162
Stx2-II	NC_004914	89	75.280	5.239
Stx2-86	NC_008464	81	74.070	5.150
cdtI	NC_009514	60	71.670	4.977
Min27	NC_010237	83	71.080	4.915
Stx2-1717	NC_011357	77	63.640	5.082

*M*_SD_ was smaller than the optimal 6 nt (Schurr *et al.*, 1993; Vimberg *et al.*, 2007) for both Clade A and Clade B phages, which simplifies our statistical analysis. That is, we only need to test whether *M*_SD_ is significantly greater in Clade A phages than in Clade B phages, which is equivalent to testing which mean *M*_SD_ is closer to the optimal *M*_SD_. The mean *M*_SD_ was greater for the eight Clade A phages (5.8930) than the 16 Clade B phages (5.0190), the difference being statistically highly significant (*t*-test assuming equal variance, *t* = 12.5160, d.f. = 22, *P*<0.0001, two-tailed test). The variance in *M*_SD_ was nearly identical between the two groups. In short, both *P*_SD_ and *M*_SD_ supported our hypothesis that translation initiation was more efficient in Clade A phages than in Clade B phages.

### Comparison of secondary structure stability between Clade A and Clade B phages

The secondary structure formed from the 40 bases upstream of the start codon may bury the SD sequence and consequently interfere with the SD–aSD pairing. Our hypothesis predicted that Clade A phages should have a weaker secondary structure (less negative MFE_40nt_) than Clade B phages. The empirical evidence strongly supported this prediction (Table 2), with MFE_40nt_ significantly weaker in Clade A phages (mean MFE_40nt_ = −5.1770) than in Clade B phages (mean MFE_40nt_ = −6.4610, *t* = 6.7879, d.f. = 22, *P*<0.0001, two-tailed test).

**Table 2.** Secondary structure stability (MFE_40nt_ and MFE_−4+37_) in Clade A and Clade B phages

Phage	GenBank accession no.	No. of coding sequences	MFE_40nt_	MFE_−4+37_
**Clade A**				
13 a	NC_011045	55	−5.3735	−4.7076
EcoDS1	NC_011042	53	−5.5532	−5.6291
K1-5	NC_008152	52	−5.2631	−3.8735
K1E	NC_007637	62	−4.8619	−4.1960
K1F	NC_007456	43	−5.8679	−6.5579
T3	NC_003298	55	−5.1473	−4.6391
T7	NC_001604	60	−5.0047	−4.2622
BA14	NC_011040	52	−4.3469	−4.2873
**Clade B**				
ϕP27	NC_003356	58	−5.7564	−5.2038
SFV	NC_003444	53	−6.5808	−6.0811
933W	NC_000924	80	−6.2833	−6.4413
Min27	NC_010237	83	−6.4707	−6.0010
VT2-Sakai	NC_000902	83	−5.9863	−6.2927
Stx2-I	NC_003525	166	−6.4645	−6.6228
BP-4795	NC_004813	85	−7.0353	−5.9579
cdtI	NC_009514	60	−7.0232	−6.1000
HK022	NC_002166	57	−6.2793	−4.6179
HK97	NC_002167	61	−6.6669	−4.7044
λ	NC_001416	73	−6.9789	−5.7668
N15	NC_001901	60	−7.0329	−5.6890
Stx1-phage	NC_004913	84	−6.0289	−5.6139
Stx2-II	NC_004914	89	−6.3666	−5.9200
Stx2-1717	NC_011357	77	−6.6052	−5.8212
Stx2-86	NC_008464	81	−5.8188	−5.5885

The secondary structure formed around the start codon may interfere with the accessibility of the start codon, which is crucially important for translation initiation in bacterial species (Nakamoto, 2006). Our hypothesis predicted that Clade A phages should have less negative MFE_−4+37_ (weaker secondary structure) at this region than Clade B phages, which is again supported by the empirical evidence (Table 2). The mean MFE_−4+37_ was −4.7690 for the eight Clade A phages and −5.7760 for the 16 Clade B phages, the difference being statistically significant ( *t* = 3.4170, d.f. = 22, *P* = 0.0025, two-tailed test). Thus, the differences in secondary structure stability between Clade A and Clade B phages were also consistent with the interpretation that genes in Clade A phages have more efficient translation initiation than those in Clade B phages.

### Relationship between SD features and secondary structure stability

If efficient translation initiation is evolutionary beneficial, then both SD features (*P*_SD_ and *M*_SD_) and the MFE of sequences flanking the start codon will be subject to the same selection and consequently are expected to have correlated changes. That is, a gene that requires high translation initiation efficiency is expected to have both high *P*_SD_ and *M*_SD_, and weak MFE_40nt_ and MFE_−4+37_. However, if there is an optimal rate of translation with a rate too high or too low being not as good, then an increase in *P*_SD_ and *M*_SD_ may result in selection increasing the stability of the secondary structure to maintain the optimal rate. In that case, we may observe a negative correlation between SD features (*P*_SD_ and *M*_SD_) and structural features (MFE_40nt_ and MFE_−4+37_).

**Fig. 2.**
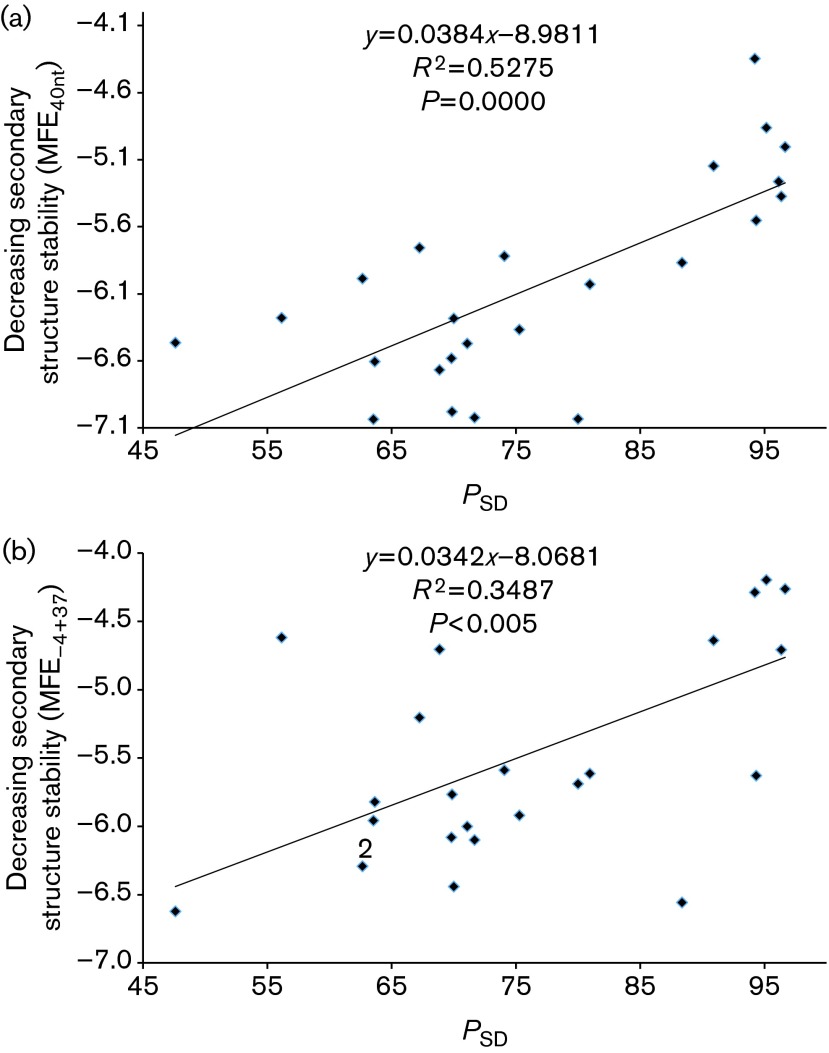
A high *P*_SD_ is associated with weak secondary structure around the SD sequence and start codon measured by MFE in *E. coli* phages at two locations: (a) 40 bases upstream of the start codon (MFE_40nt_) and (b) from four bases upstream to 37 bases downstream of the start codon (MFE_−4+37_).

**Fig. 3.**
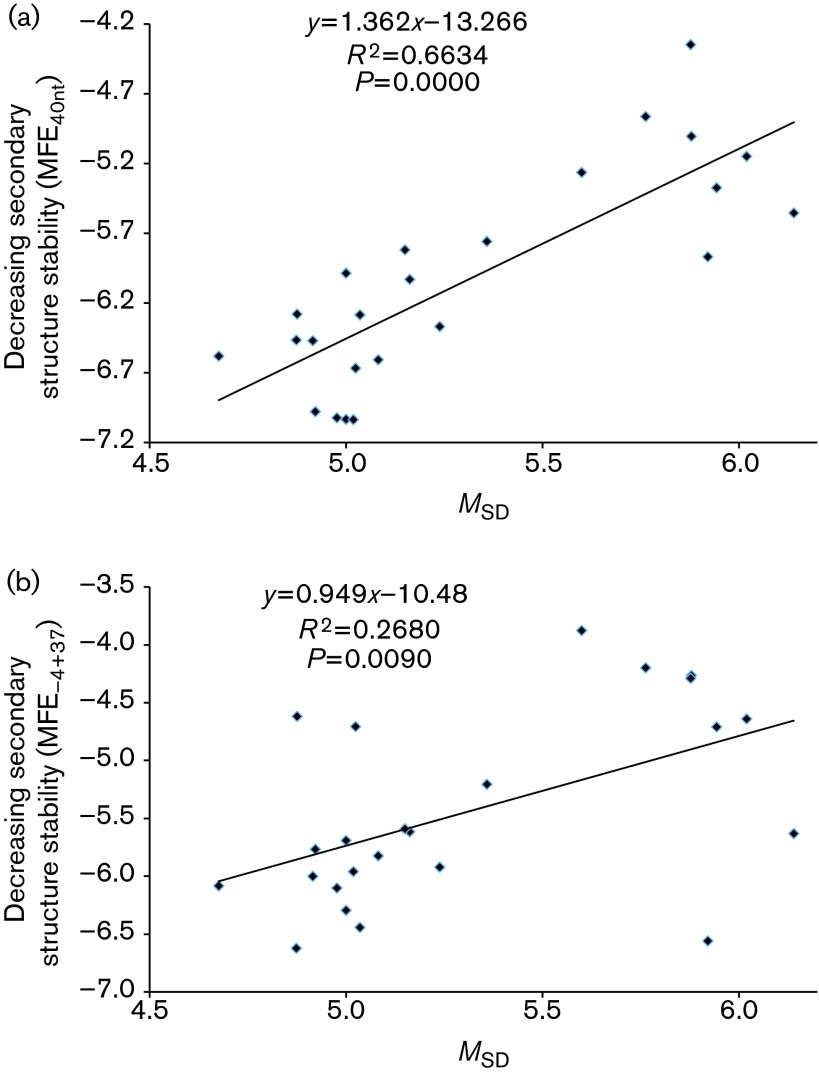
A large *M*_SD_ (strong SD with more base pairs with aSD) is associated with weak secondary structure around the SD sequence and start codon measured by MFE in *E. coli* phages at two locations: (a) 40 bases upstream of the initiation codon (MFE_40nt_) and (b) from four bases upstream to 37 bases downstream of the initiation codon (MFE_−4+37_).

We observed a highly significant positive correlation between *P*_SD_ and two secondary structure features (MFE_40nt_ and MFE_−4+37_, Fig. 2). A strong positive correlation was also observed for *M*_SD_ and the two MFE measures (Fig. 3). This suggested selection operating to maximize translation initiation efficiency in phages instead of stabilizing it at one particular level.

As some phage species share a common ancestry, a more appropriate characterization of the relationship between SD features (*P*_SD_ and *M*_SD_) and secondary structure features (MFE_40nt_ and MFE_−4+37_) should be carried out with the method of phylogeny-based independent contrasts (Felsenstein, 1985). We performed the contrasts using dambe (Xia, 2013b),which implemented the method with extensions (Xia, 2013a) and the tree from a previous study (Chithambaram *et al.*, 2014b). The four positive associations (Figs 2 and 3) were still significant (*P*<0.05).

### Virulent phages exhibit better translation adaptation than temperate phages

Our second objective was to understand why genes in Clade A phages exhibited better adaptation in translation initiation and elongation than those in Clade B phages. All eight coliphages in Clade A were virulent and all 16 coliphages in Clade B were temperate, suggesting phage lifestyle as a contributing factor. Virulent phages are almost always engaged in translation once they enter the host cell. In contrast, protein-coding genes in a prophage are under little purifying selection and the evolutionary success of temperate phages may not necessarily rely on rapid biosynthesis. Thus, selection for more efficient translation may be stronger in the virulent phages than in temperate phages, leading to more efficient translation initiation and better codon adaptation in virulent phages (Clade A) than temperate phages (Clade B). This hypothesis was consistent with coliphages, not only for phages in Clades A and B, but also for the other 28 phages in fig. 8 of Chithambaram *et al.* (2014b) not included in Clades A and B. To further test the generality of this lifestyle hypothesis, we analysed 35 phages (six virulent and 29 temperate) infecting the Gram-positive *Staphylococcus aureus*.

There are notable differences between the two host species that are relevant to understanding phage adaptation. First, *S. aureus* genes tend to have SD sequences more often than *E. coli* genes. If we operationally define a SD sequence as a sequence (1) of at least 4 nt long, (2) located within 30 nt upstream of the initiation AUG and (3) having perfect base-pairing with the last 13 nt at the 3′ end of 16S rRNA, then *P*_SD_ is significantly higher (χ^2^ = 136.69, d.f. = 1, *P*<0.0001) in the 2767 *S. aureus* genes (0.9256) than in the 4321 *E. coli* genes (0.8287). Second, the secondary structure of the sequences flanking SD and start codon is significantly weaker in *S. aureus* genes than in *E. coli* genes, with mean MFE_40nt_ equal to −2.9000 for *S. aureus* and −5.5543 for *E. coli* (*t* = 35.8074, d.f. = 7086, *P*<0.0001), and mean MFE_−4+37_ equal to −3.1422 for *S. aureus* and −4.9700 for *E. coli* (*t* = 28.0094, d.f. = 7086, *P*<0.0001). This suggested that the translation machinery in *S. aureus* had a more stringent requirement for the 5′ end of mRNA than *E. coli*.

The *Staphylococcus* phages, both virulent and temperate, had high *P*_SD_ values similar to their host, but did not differ either in *P*_SD_ or *M*_SD_ between the six virulent and the 29 temperate phages. However, there were significant differences in MFE_40nt_, MFE_−4+37_ and codon adaptation measured by *I*_TE_ between virulent and temperate *Staphylococcus* phages in the predicted direction. The secondary structure flanking the SD sequence was weaker in virulent *Staphylococcus* phages than in temperate phages, with mean MFE_40nt_ being −2.9494 in the former and −3.3560 in the latter (*t* = 3.0723, d.f. = 33, *P* = 0.0042, two-tailed test). Similar differences were observed in sequences flanking the start codon, with mean MFE_−4+37_ being −2.5402 in the former and −2.8792 in the latter (*t* = 2.6714, d.f. = 33, *P* = 0.0116, two-tailed test).

**Fig. 4.**
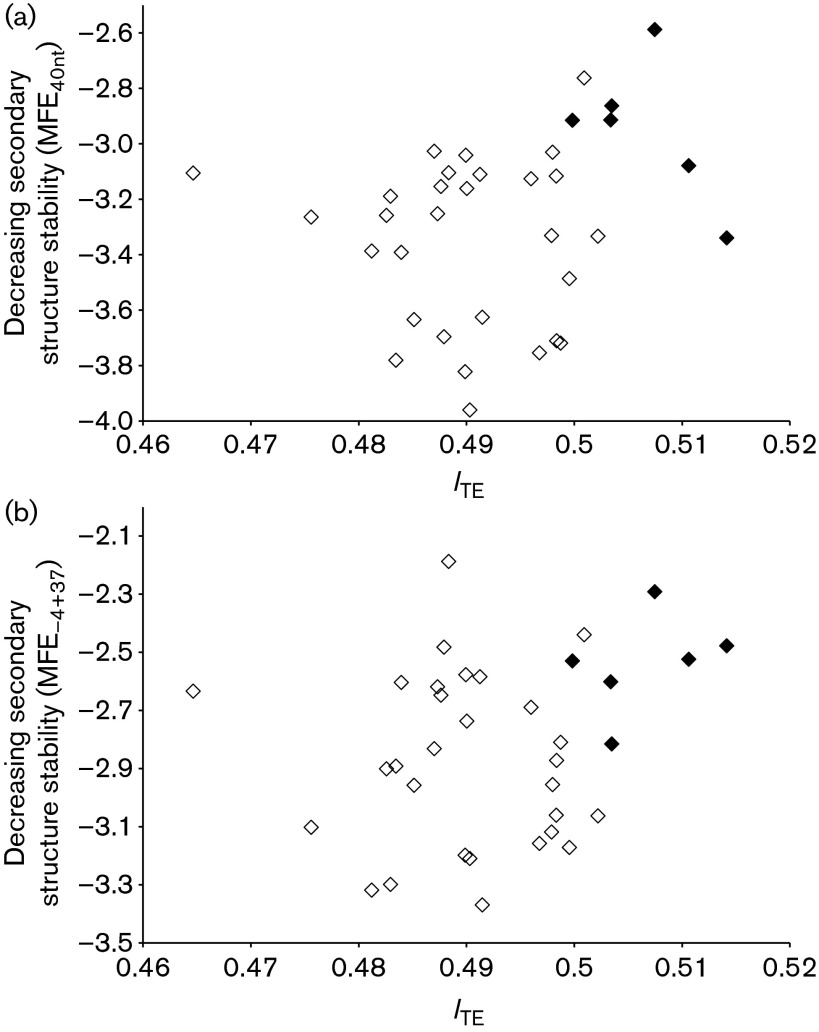
Virulent *Staphylococcus* phages (♦) have genes with relatively weaker secondary structure in sequences flanking the SD sequence and start codon as well as better codon adaptation to the host than temperate phages (⋄). (a) MFE_40nt_. (b) MFE_−4+37_.

For measuring codon adaptation, we used *I*_TE_ which has the advantage over CAI in that *I*_TE_ incorporates background mutation bias (Xia, 2014). *I*_TE_ is computed by four different methods in dambe (Xia, 2013b) that differ in treating synonymous codon families, and we use the second method that breaks compound synonymous codon families (e.g. sixfold Arg, Ser and Leu for the standard code) into two separate four- and twofold codon families. *I*_TE_ was significantly greater in the virulent *Staphylococcus* phages than in the temperate phages, with the mean *I*_TE_ being 0.5065 for the former and 0.4899 for the latter (*t* = 11.1861, d.f. = 33, *P*<0.0001, two-tailed test). As shown in Fig. 4, high *I*_TE_ values of the coding sequences in virulent *Staphylococcus* phages were associated with weak secondary structure at the 5′ end of mRNA, measured by MFE_40nt_ and MFE_−4+37_.

## Discussion

Evolution of codon usage and translation elongation efficiency has recently been recognized to depend on translation initiation efficiency (Supek & Šmuc, 2010; Tuller *et al.*, 2010; Xia, 2014; Xia *et al.*, 2007). In short, an mRNA with low translation initiation efficiency is not expected to increase protein production with optimized codon usage. In contrast, protein production for an mRNA with high translation initiation efficiency may become limited by translation elongation and such an mRNA can increase protein production with optimized codon usage. This implies little selection for codon optimization for genes with low translation initiation efficiency, but strong selection for codon optimization for genes with high translation initiation efficiency.

We have extended this hypothesis to explain why two clades of *E. coli* phages differ greatly in codon adaptation to their hosts (Chithambaram *et al.*, 2014b). In particular, why Clade A phages exhibit stronger codon adaptation than Clade B phages. Our hypothesis that genes in Clade A phages have higher translation initiation efficiency than those in Clade B phages is highly consistent with our empirical results (Tables 1 and 2), with the Clade A phages having both a higher *P*_SD_ and *M*_SD_ than the Clade B phages. Higher *P*_SD_ has also been observed in highly expressed genes than lowly expressed genes in *E. coli* (Ma *et al.*, 2002).

Our finding of a positive correlation between strong SD sequences and weak secondary structure (Figs 2 and 3) suggests that natural selection may operate simultaneously to optimize these features to increase translation initiation efficiency. One may suggest that the presence of a SD sequence, which is typically purine-rich, may itself result in a change in the two MFE measures, so that the positive correlations in Figs 3 and 4 have little to do with simultaneous selection on both SD features and secondary structure features. This suggestion is not true. If we replace a 6mer in the sequences flanking the start codon by a typical SD sequence such as AGGAGG, the resulting MFE_40nt_ and MFE_−4+37_ may increase or decrease, but overall do not become significantly weaker. Thus, the presence of a stronger SD sequence in the Clade A phage genes cannot explain its weaker MFE_40nt_ and MFE_−4+37_.

Given the seemingly obvious benefit of efficient translation, one would naturally ask what has prevented the Clade B phages from acquiring more efficient translation initiation, i.e. higher *P*_SD_ and *M*_SD_ and weaker MFE_40nt_ and MFE_−4+37_. As we have mentioned earlier, both clades have evolved and diverged into multiple lineages in the *E. coli* host, so lack of evolutionary time may not be the right answer.

Our results are consistent with the lifestyle hypothesis. That is, selection for more efficient translation may be stronger in the virulent phages than in the temperate phages, leading to more efficient translation initiation and better codon adaptation in the virulent (Clade A) phages than the temperate (Clade B) phages. Whilst this interpretation of phage lifestyle on translation is consistent with the coliphages, we have shown the interpretation is also consistent with the *Staphylococcus* phages (six virulent and 29 temperate phages). The secondary structure of sequences flanking the SD sequence and start codon is significantly weaker in the virulent than in the temperate *Staphylococcus* phages (Fig. 4). The *I*_TE_ is also highly significantly greater in the virulent than in the temperate *Staphylococcus* phages (Fig. 4).

A previous study (Chithambaram *et al.*, 2014a) used correlation in relative synonymous codon usage (*r*_RSCU_) between phage and host as a measure of phage codon adaptation, and found temperate phages to have higher *r*_RSCU_ values than virulent phages. This seems to contradict the conclusion that Clade A phages (all being virulent) exhibit better codon adaptation than Clade B phages (all being temperate). However, *r*_RSCU_, like the effective number of codons (Sun *et al.*, 2013; Wright, 1990), is strongly affected by mutation bias when RSCU for the host is computed from all *E. coli* genes. When host highly expressed genes are used for computing RSCU, the difference in *r*_RSCU_ between the virulent and temperate phages becomes smaller. If one uses *w_i_* (equation 2 in Xia, 2014) from the host as host RSCU (*w_i_* is essentially RSCU corrected for mutation bias), then *r*_RSCU_ is significantly greater for the virulent phages than for the temperate phages.

Another relevant observation is that all eight phage species in Clade A have all their genes on the same DNA strand and all 16 phage species in Clade B have their genes distributed on both DNA strands. Strong strand asymmetry can affect both synonymous and non-synonymous substitutions (Chithambaram *et al.*, 2014b; Marín & Xia, 2008; Xia, 2012b, c). If two DNA strands have dramatically different mutation bias, then mutation bias in one strand that is in the same direction as codon adaptation is necessarily accompanied by mutation bias in the other strand going against codon adaptation.

## Methods

### 

#### Genomic data.

The genomes of *E. coli* and *S. aureus*, as well as their phages, were retrieved from GenBank. Coding sequences were extracted and their codon usage analyzed by dambe (Xia, 2013b). Only coding sequences with at least 33 codons were included to alleviate stochastic fluctuations of codon usage. All phage genomes were scanned for tRNAs by using the tRNAscan-SE Search Server (Schattner *et al.*, 2005). Phage data compilation consisting of clade, phage name, phage family, phage GenBank accession number, phage genome length, number of coding sequences in each phage genome, *I*_TE_ and the number of tRNA genes encoded in each phage genome are included in Table S1 (available in the online Supplementary Material).

#### Identification of SD sequences.

**Fig. 5.**
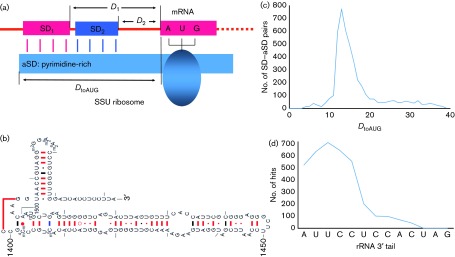
(a) Schematic representation of the SD sequence on mRNA pairing with the aSD sequence on the SSU rRNA. (b–d) The free 3′ end of SSU rRNA (b), the frequency distribution of 4577 putative matches of at least four bases between the rRNA 3′ tail and the upstream 30 nt of coding sequences (c), and the number of times each nucleotide site at the rRNA 3′ tail participated in the SD–aSD matches (d).

We also extracted 30 nt upstream of the start codon (Upstream30) from each gene in phage and host genomes, and the last 20 nt of the *E. coli* small subunit (SSU) rRNA using dambe (Xia, 2013b), to identify SD sequences. As we show below, it is not appropriate to define the SD sequence simply as an AGGAGG motif within a fixed distance range upstream of the start codon. The SD sequence on the mRNA and the aSD sequence on the SSU rRNA pair to position the anticodon of the initiation tRNA at the start codon (Fig. 5a). The optimal location of the SD sequence in the literature is often measured by the distance from the SD sequence to the start codon (e.g. *D*_1_ and *D*_2_ in Fig. 5a) or from the middle of the SD sequence to the start codon (Osterman *et al.*, 2013). However, this approach is probably incorrect as illustrated in Fig. 5(a). Both SD_1_ and SD_2_ position the tRNA anticodon properly at the start codon AUG, but their associated *D*_1_ and *D*_2_ are different (Fig. 5a). A correct distance measure should take into consideration the relative position of both mRNA and the rRNA 3′ tail. One such distance is the distance from the end of the SSU rRNA to the beginning of the start codon (*D*_toAUG_, Fig. 5a).

Based on the *E. coli* SSU rRNA secondary structure (Woese *et al.*, 1980; Yassin *et al.*, 2005), there are 13 nt at the 3′ end of the rRNA (referred to as the rRNA 3′ tail hereafter) that are free to base-pair with the SD sequence (Fig. 5b). We searched each Upstream30 sequence against the rRNA 3′ tail for matches with a length of at least four consecutive bases. The frequency distribution of *D*_toAUG_ from 4577 such matches peaked at *D*_toAUG_ = 13 and decreased rapidly towards *D*_toAUG_ = 10 and *D*_toAUG_ = 20 (Fig. 5c). We thus operationally defined a SD sequence as a sequence four bases or longer that can pair with the rRNA 3′ tail leading to a *D*_toAUG_ within the range of 10–20. Note that a SD sequence such as AGGAGG would need a space of five bases between the end of the SD sequence and the beginning of the start codon in order to have a *D*_toAUG_ = 13. A SD sequence such as AGGAG would need to have six bases between the end of SD and the beginning of the start codon in order to have a *D*_toAUG_ = 13.

Although the rRNA 3′ tail has 13 bases free (Fig. 5b), the sites that are involved in SD–aSD base pairing mainly belong to the first six sites (Fig. 5d). However, 754 putative SDs (including 156 GUGA, 166 GAGGU, 169 AGGU and 263 UGAU) in Upstream30 sequences in *E. coli* genes involve the second A from the 3′ end of SSU rRNA. This is consistent with the experimental observation that mutations at that site are moderately deleterious (Yassin *et al.*, 2005).

We computed two indices for each phage: (1) percentage of SD-containing genes (*P*_SD_) and (2) mean number of consecutively matched sites (*M*_SD_). Previous studies have shown that highly expressed *E. coli* genes are more likely to have a SD sequence than lowly expressed genes (Ma *et al.*, 2002) and that *M*_SD_ is important for gene expression (Osterman *et al.*, 2013).

#### Measuring stability of local mRNA secondary structure.

The stability of local secondary structure formed in mRNA is generally measured by MFE (kJ mol^−1^). The more negative the MFE value, the greater the stability of the secondary structure. We computed MFE using dambe, which implements the functionality of the Vienna RNA package (Hofacker, 2003). The settings used were: folding temperature 37 °C, with no lonely pairs and with no G/U pairs at the end of helices. Changing these settings did not affect the relative magnitude of MFE.

Translation initiation greatly depends on the secondary structure of sequences flanking the start codon (de Smit & van Duin, 1990, 1994; Nivinskas *et al.*, 1999; Xia & Holcik, 2009; Xia *et al.*, 2011). Burying either the SD sequence or the start codon in a stable secondary structure would affect its accessibility and decreases protein production dramatically in *E. coli* (Osterman *et al.*, 2013). For this reason we measured the stability of the secondary structure for two associated regions: (1) 40 bases upstream of the start codon where the presence of a hairpin strongly inhibits translation (Osterman *et al.*, 2013), and (2) the region −4 to +37, which has been previously studied and considered as a key contributor to translation initiation (Kudla *et al.*, 2009; Osterman *et al.*, 2013; Xia, 2014). MFEs for the two regions were designated MFE_40nt_ and MFE_−4+37_, respectively. The two regions are related, respectively, to the accessibility of the SD sequence and the start codon.

#### Phage lifestyle classification.

The classification of phages into temperate and virulent categories was based on three publications (Deschavanne *et al.*, 2010; Lima-Mendez *et al.*, 2007; McNair *et al.*, 2012). For *E. coli* and *S. aureus* phages, phage name, family, GenBank accession number of their sequenced genomes and their lifestyle identification (virulent/temperate) are available as Tables S2 and S3.
